# Hypersensitivity reactions due to black henna tattoos and their components: are the clinical pictures related to the immune pathomechanism?

**DOI:** 10.1186/s12948-017-0063-6

**Published:** 2017-04-10

**Authors:** Gianfranco Calogiuri, Elisabetta Di Leo, Lavjay Butani, Stefano Pizzimenti, Cristoforo Incorvaia, Luigi Macchia, Eustachio Nettis

**Affiliations:** 1 Pneumology and Allergy Department, Hospital Sacro Cuore, Gallipoli, Lecce, Italy; 2 Section of Allergy and Clinical Immunology, Unit of Internal Medicine-“F. Miulli” Hospital, Acquaviva delle Fonti, Bari, Italy; 3 Department of Pediatrics, University of California Davis School of Medicine, Sacramento, USA; 4 Allergy Outpatients’ Clinic, ASL-TO3, Ospedale Civile “E. Agnelli”, Pinerolo, Turin, Italy; 5 ASST Specialistic Center Gaetano Pini/CTO, Milan, Italy; 6 Section of Allergy and Clinical Immunology, Department of Internal Medicine and Infectious Diseases, University of Bari Medical School, Bari, Italy

**Keywords:** Para-phenylenediamine, Henna tattoo, Black henna, Contact dermatitis, Allergy

## Abstract

Hypersensitivity to para-phenylenediamine (PPD) and related compounds induced by temporary black henna tattoos has become a serious health problem worldwide. Different patterns of sensitization with various clinical aspects are described in literature due to PPD associated to henna tattoo and these manifestations are likely correlated with the immunological and dermatological pathomechanisms involved. Henna is the Persian name of the plant *Lawsonia inermis,* Fam. *Lythraceae*. It is a woody shrub that grow in regions of North Africa, South Asia, India and Sri Lanka. Nowadays it is rather frequent to see temporary “tattoos” performed with henna. To make tattoos darker and long-lasting PPD has been associated to henna in tattoo drawings mixtures, so obtaining “*black henna*”. In these years there has been a rise of contact sensitization to PPD and in medical literature an increased number of cases have been reported on temporary henna tattoo application. Here we review the various clinical patterns related to PPD and henna tattoo, to investigate the possible link between clinic-morphological pictures and the immunological response to PPD and henna. The literature underlines that different clinical manifestations are related to black henna containing PPD, and its derivative products may cause delayed-type as well as immediate-type reactions. Further studies are needed to investigate the relationship between clinical and morphological aspects of PPD contact dermatitis and the T cell subsets predominance.

## Background

The term “tattoo” derives from two Polynesians words, “TA”, which means “to draw” and “TOO” or “TAU” which means “spirit”. The whole word means “draw the body”. The tattooing procedures have been quite variable in different ages, depending on the cultures involved and thus influencing the perception of a positive or a negative significance. The Hebrews follow Torah exhortations (“you won’t make any incision on your body”, Deuteronomy, 14 but also Leviticus, 19:28) while for the Maoris the tattoo drawings on the face indicate the social class and status, the profession, the clan and the ability in a battle [1].

Nowadays, in a “liquid society” [2], where nothing is forever, it has become rather frequent to see temporary “tattoos” performed with henna. Painless, highly decorative and, above all, temporary, the henna colour lasts for up to 3 weeks, making it is the perfect way of creating a fashionable look while avoiding the consequences or risks connected to a permanent tattoo.

Henna is the Persian name of the plant *Lawsonia inermis,* Fam. *Lythraceae*. It is a woody shrub, reaching heights up to 3 metres, which grows up mainly in the warm, moist regions of North Africa, South Asia, India and Sri Lanka. Henna plant was identified by Linnaeus, who called it with that name to honour the Scottish doctor of his scientific expedition, Isaac Lawson. The active dying ingredient of henna is a naphtoquinone called *lawsone* (2-hydroxy-1,4-naphthoquinone), constituting about 1% by weight of the crushed leaves [3].

Commercially, henna is available as a green powder, obtained from crushing dried leaves mixed with water and oil to create a paste. Natural henna gives a brownish-orange pigment to the skin when applied (*red henna*), but the colour will darken to brown. Various substances, such as lemon oil, vinegar, eucalyptus oil and coffee, may be added to obtain different colouring effects and a thick paste [4]. The henna is left on the skin to dry for at least 2 h and preferably up to 24 h. The longer henna is put in contact with the skin, the darker the resulting colour will be [3]. As far as allergic reactions are concerned [5, 6], the pure henna is a weak sensitizer and it is poorly responsible for allergic contact dermatitis [7–9], but when para-phenylenediamine (PPD), that is a strong sensitizing agent, is added the frequency of allergic reactions increases. PPD is responsible for professional contact hypersensitivity in photographers, hairdressers and workers manipulating rubber components [10]. PPD can induce different types of hypersensitivity reactions, which may be framed from different points of view, according to the time-pattern onset or according to their morphological aspects.

## Methods

We reviewed the available literature concerning the clinical presentations, the morphological pictures associated to allergy to black henna and the immunological mechanisms underlying the allergic reactions.

## Results

### Clinical manifestations related to a time-pattern onset

Generally, the first signs of allergic contact dermatitis caused by black henna tattoos develop within 1–3 days in those already sensitized, and within 4–14 days in patients who become sensitized by the tattoo [5]. In 2002 Chung et al. suggested that patients sensitized to black henna tattoo should be divided into 2 groups according to their clinical course, that may be acute or subacute. This classification was based on the previous studies of Buckley on photographers [11].

Thus, the clinical features define 2 groups: an acute response to temporary tattooing, typically presenting with intense eczematous responses within 1–2 days of tattooing, and a subacute response, that is, developing lichenoid eruptions slowly in 1–2 weeks [11].

Some reactions may appear 45 days later following the henna tattoo application [12], but sometimes cutaneous reactions may appear few hours following henna tattoo [13, 14]. The latter possibility is caused by a pre-existing sensitization to PPD, probably due to a hidden exposure to cross-reactive compounds belonging to the para-amino group substances [15].

For this reason, beyond the 2 groups identified by Chung et al. there is a third group of patients, whose symptoms onset occurs few hours after a clear exposure to PPD. Their clinical history often includes a previous black henna tattoo, performed months or years ago, which behaves like a silent promoter of sensitization to PPD.

In patients with a silent sensitization to PPD, localized cutaneous manifestations are usually represented by a severe inflammatory dermatitis leading to skin ulcerations following a further henna tattoo [14, 15], but even by a contact urticaria [16] or a contact anaphylaxis due to henna tattoo [17] or henna hair dye [18].

Such clinical expressions are based on a IgE-mediated reaction to vegetable components of henna, and particularly specific IgE towards a 25 kDa henna protein have been detected by immunoblotting [19]. However, when applied as a hair dye, PPD alone with no henna components has been responsible for immediate-type contact anaphylaxis [20–22] or for delayed-type anaphylactic reaction [23], rarely even with a fatal outcome [24].

Another kind of hypersensitivity reactions to PPD is represented by a severe facial angioedema, appearing few hours after a dark hair painting in patients who have previously performed a black henna tattoo [25–27]. This is an acute, severe and even life-threatening condition, sometimes requiring an admission in an Intensive Care Unit [25, 28, 29], because of the involvement of the upper airways frequently in young patients [30–32].

Interestingly, people poisoned by PPD ingestion for suicidal purposes [33, 34] or intoxicated by an extensive black henna tattoo application [35, 36] or by a hair dye [36, 37] show clinical signs of rhabdomyolysis, anemia and acute renal failure always associated to a severe angioneurotic oedema of the upper airway accompanied by a swollen, dry, hard, and protruding tongue.

### Morphological clinical pictures

As far as morphological clinical pictures are concerned, they appear to be more various and heterogeneous than time-pattern contact allergy manifestations (Table 1). Thus, beyond the aforementioned urticaria and angioedema or contact anaphylaxis related to black henna tattoo painting and the classical eczematous contact dermatitis [38, 39], have been described lichenoid eruptions [11, 40, 41], lymphomatoid reactions [42, 43], vesicular-bullous lesions [44], which may be very painful and invalidating [45, 46], and erythema multiforme like eruptions [29, 47]. Sometimes, the localized eruption may become generalized and associated to systemic symptoms as fever and malaise [50, 51]. Exceptionally, a Sweet syndrome [52], Wells syndrome [53, 54], prurigo nodularis [55] and connubial contact dermatitis [56, 57] have been also described.

**Table 1:** Reactions to black henna tattoo: main clinical manifestations and time to onset after the application

Clinical manifestation	Time to onset	Ref
Eczematous contact dermatitis	1–2 days	[11, 49, 50]
Lichenoid eruptions	1–2 weeks	[11, 51, 52]
Contact urticaria and angioneurotic oedema	Few hours–2 days	[19, 33, 34]
Contact anaphylaxis	Few hours	[20]
Lymphomatoid reactions	Few hours–few days	[53, 54]
Vesicular-bullous lesions	1–4 days	[55, 56]
Erythema multiforme like eruptions	2–14 days	[58–60]
Sweet syndrome	3 days	[63]
Wells syndrome	3 days	[64, 65]
Prurigo nodularis	3 months	[66]
Connubial contact dermatitis	Few days	[67, 68]

It appears obvious that PPD is able to induce different clinical and morphological pattern as it happens in T-cell mediated drug hypersensitivity reactions [58], although PPD is a contact allergen only. Wolkenstein et al. suggested that detoxification enzymatic pathway defects are involved in toxic epidermal necrolysis and other severe cutaneous pathogenesis, allowing reactive metabolites accumulation for drugs as sulfonamides and anticonvulsant [59].

Because keratinocytes exert metabolic activity with two N-acetyltransferases (NAT) 1 and 2 (NAT2), isoenzymes for PPD in the skin [60], in a further in vitro investigation it has been observed that genotyping for NAT1 and NAT2 polymorphisms performed in 147 PPD-sensitized patients, the rapid acetylator NAT1*10 allele were under-represented in PPD-sensitive patients compared with 200 control subjects [61]. Such study suggests PPD slow acetylators NAT2 patients are more prone to develop an erythema multiforme-like reaction [47–50], i.e. a more serious contact allergy, as well as drug induced severe cutaneous eruptions are more frequently observed in slow acetylators [59]. However, in patients with severe cutaneous drug reactions, an expansion of drug specific cytotoxic T CD8+ cell subset and infiltration in cutaneous lesions has been observed in the immuno-histopathologic specimens [62]; T cells subsets and different cytokine patterns are involved in the variability of clinical pictures in drug induced hypersensitivity reactions [58].

By comparing the available data, PPD seems to be a hapten with a peculiar aspect, because it may behave like a systemic drug to elicit a hypersensitivity response. Actually, in PPD sensitized patients specific PPD T cell clones CD4+ CD8+ secreting Th2 cytokine pattern, as IL-4, IL-5, IL-6, IL-8, IL-10, and IL-13, have been identified and isolated in vitro [63, 64]. This was reported also in mice, because PPD-HSA causes a transient activation of CD8+ cytotoxic cells only [63]. However, because vesicular bullous reactions have been reported in patients with contact hypersensitivity to PPD after henna tattoo application [44–46] and two other patterns of T cell activation by PPD have been evidenced in vitro [63], future researches should investigate the relationship between clinical morphological aspect of PPD contact dermatitis and T-cell subsets predominance.

## Discussion and conclusions

Henna has been used traditionally for medicinal purposes and as a cosmetic. For medicinal purposes, it has been used as a bitter tea for stomach or intestinal problems, for fever and headache, as a paste to cure ringworm or nail fungus, to reduce chafing and prevent blisters and to soothe irritated, dry or chapped skin [3]. As a cosmetic, henna has been used as a hair thickener, as a nail colorant and conditioner and as a decorative stain for the body. Also, henna has been used as a brown dye for wool, cotton, and silk [3]. Moreover, henna has great significance in all Eastern wedding traditions, and no wedding is complete without drawing floral motifs or geometric decorations on the bride’s hands and feet. The increasing use of henna in the 2000s to make temporary tattoos raised the issue of allergic reactions.

Actually, as reported above, the pure henna is a weak sensitizer, but the addition of PPD to make tattoos darker and long-lasting, thus obtaining *black henna,* using mixtures that are often extemporaneously prepared with a variety of materials and sources and very variables concentrations of PPD resulted in a recent exploit of contact sensitization to PPD. In fact, an increased number of cases have been reported through temporary henna tattoo application. Therefore, dermatologists, paediatricians and allergists have alerted about the rising frequency of black henna contact allergy [5, 6]. We reviewed the various clinical patterns related to PPD and henna tattoo to investigate the possible link between clinic-morphological pictures and the immunological response to PPD and henna. The main results may be summarized in a clinical expression with different manifestations related to black henna containing PPD, and in a spectrum of allergic responses including both delayed-type and immediate-type reactions.

Fadden et al. had previously demonstrated that the allergenic component of PPD accumulates in the skin. Hence, intermittent exposure to low concentration of PPD may be equivalent to a higher concentration in a one-off exposure [65]. Because different studies, performed in USA and in United Arab Emirates, have shown that the presence of PPD high concentrations in henna dye mixture ranges from 2 to 29.5% measured through high performance liquid chromatography method [5, 66, 67], it is not unusual that a single henna tattoo, performed as a summer caprice, may be sufficient to induce a silent or clear PPD sensitization.

Actually, no specific IgE to PPD or any related oxidative product have been identified in vitro, but it has been demonstrated that a radio-labelled PPD containing oxidative hair dye applied to human volunteers for 30 min is eliminated predominantly (80–90%) by renal excretion, being accumulated in urine [68]. Such study showed that there is a systemic absorption of PPD through the skin. Goldberg et al. had previously reported that the oxidation product of PPD causing the immediate-type reaction is N′N′-bis(4-aminophenyl)-2,5-diamino-1,4-quinone-dimine [69], also known as Brandowski base (BB), although they were unable to produce a suitable RAST assay [69]. A further investigation has recently shown that, once penetrated in human body, PPD oxidative products bind irreversibly to cysteine at 34 position on human serum albumin [63], so turning to a complete antigen, but it is unclear whether IgE are directed towards the chemical component only or towards the chemically modified albumin which has become immunogenic. Actually, such an IgE-mediated mechanism has been described only in ethylene oxide hypersensitivity [70].

In suggestive clinical pictures, like the urticarial-angioedema-like contact dermatitis, although the mechanisms appears as an IgE-mediated reaction, actually it is an early onset cell-mediated mechanism [26, 27]. It has been suggested, from experimental studies on animals, that specific T-cells with a Th2 cytokines pattern profile specific to PPD could be the effectors cells of delayed response [71]. However, in vitro studies have suggested that also in humans there is a recruitments and activation of Th2 clones in response to PPD exposure [63].

Padovan et al., in their studies on hypersensitivity to beta-lactams antibiotics, reported that Th1 profile, very common in contact allergy, may shift to a Th2 profile depending on the hapten concentration [72]. Because high concentrations of PPD are present in henna tattoo paintings [16, 67], it is possible that PPD contact dermatitis is mediated by specific Th2 cell clones, secreting interleukin (IL)-4 and IL-5. Such a pathomechanism has been confirmed in vitro by the study of Jenkinson et all [63]. To determine whether PPD–HSA was antigenic, these authors assayed the activity of PPD and PPD–HSA on T-cell proliferation, using the lymphocyte transformation test (LTT) and the proliferation and cytokine secretion profile of CD4+ , CD8+ , and CD4/8 double-positive T-cell clones isolated by stimulating T cells from the blood of allergic volunteers with PPD or PPD–HSA. The investigators confirmed that PPD was active in the LTT and that it stimulated CD4+ , CD8+ , and CD4/8 double-positive T-cell clones with a Th2 profile [63].

Moreover, Pichler et al. elaborated the “pharmacological-immune interaction concept” (p-i concept) as they found that some drugs like sulfamethoxazole and lidocaine can bind directly to the HLA peptide complex stimulating T lymphocytes through an HLA restricted processing and metabolism independent pathway [73].

Some in vitro studies have confirmed that such immunological model is working not only for drugs, but even for some contact allergen like PPD [74]. For this reason, in case of direct exposure to high concentration of PPD, the immune response may happen through the “p-i concept” pathways, while the presence memory T-cell clones CD4+ CD45RO+, previously demonstrated in vitro [38], may accelerate the Th2 immune response in patients undergoing to hair dye painting [25–32].

However, further studies are needed to investigate the relationship between clinical and morphological aspects of PPD contact dermatitis and the T-cell subsets predominance.

These observations confirm that the pure henna is a weak sensitizer poorly responsible for allergic contact dermatitis, while PPD represents a strong sensitizing agent responsible for contact hypersensitivity. PPD can induce hypersensitivity reactions involving various pathogenetic mechanisms that are responsible for the time-pattern onset and for their morphological aspects.

## Abbreviations

PPD: paraphenylenediamine
NAT: N-acetyltransferases
